# Imaging viscosity of intragranular mucin matrix in cystic fibrosis cells

**DOI:** 10.1038/s41598-017-17037-2

**Published:** 2017-12-01

**Authors:** Sebastian Requena, Olga Ponomarchuk, Marlius Castillo, Jonathan Rebik, Emmanuelle Brochiero, Julian Borejdo, Ignacy Gryczynski, Sergei V. Dzyuba, Zygmunt Gryczynski, Ryszard Grygorczyk, Rafal Fudala

**Affiliations:** 1 University of North Texas Health Science Center, Department of Microbiology, Immunology & Genetics, Fort Worth, TX 76107 USA; 2 Centre de Recherche du CHUM (CRCHUM), Université de Montréal, Montréal, Québec, H2×0A9 Canada; 3 Texas Christian University, Department of Chemistry and Biochemistry, Fort Worth, TX 76129 USA; 4 Département de Médecine, Université de Montréal, Montréal, Québec, H3T 1J4 Canada; 5 Texas Christian University, Department of Physics and Astronomy, Fort Worth, TX 76129 USA; 6 Present Address: visiting graduate student from Moscow State University, Moscow, Russia

## Abstract

Abnormalities of mucus viscosity play a critical role in the pathogenesis of several respiratory diseases, including cystic fibrosis. Currently, there are no approaches to assess the rheological properties of mucin granule matrices in live cells. This is the first example of the use of a molecular rotor, a BODIPY dye, to quantitatively visualize the viscosity of intragranular mucin matrices in a large population of individual granules in differentiated primary bronchial epithelial cells using fluorescence lifetime imaging microscopy.

## Introduction

Mucus forms a sticky, gel-like layer that covers wet surfaces of various organs and tissues, including airways. Under physiological conditions, mucus layer act as the first line of defense against toxins and pathogens inhaled into the lungs. It is produced by the secretion and hydration of gel-forming mucins, which are large fibrous biopolymers that are synthetized and stored as a condensed matrix inside the secretory granules in mucus/goblet cells. However, under pathological conditions, such as in cystic fibrosis (CF), abnormally viscous and sticky mucus obstructs the lungs, harbors bacteria and particulates, and is not cleared by the mucociliary system, thus leading to chronic respiratory infections, progressive lung damage, and ultimately mortality^1,2^. Mucus abnormalities in CF are mostly attributed to dehydration or acidification of extracellular surface fluid into which mucins are secreted and undergo swelling and hydration^3–5^. However, it is also possible that CF mucus defects may already be present prior to mucin secretion during the early stages of biogenesis, which could impact the packaging and rheological properties of the intragranular mucin matrix. This notion has not been explored to date, and it would require the ability to assess the rheological properties of the intragranular mucin matrix.

The rheological properties of secreted mucus critically affect its physiological and pathological functions, and as a result, the viscosity of mucus has received considerable attention in an attempt to elucidate the relationship to the progression of various mucus-related disorders. It was shown that the viscosity of secreted mucus depends on a variety of environmental factors, and it varies over a wide range, from viscous fluid to gel-like states^6–8^. Standard methodologies to measure the physicochemical properties of mucus rely on the use of bulk samples of secreted mucus and classical macro-rheological techniques, such as plate rheometry, capillary viscometry, and magnetic microrheometry^5^. More recently, particle-tracking microrheology was used to characterize mucus properties with low volume samples^5^. On the other hand, the contribution of defects intrinsic to stored mucin granules, which may manifest as abnormal intragranular mucin matrix packaging and viscosity, remain unexplored. The assessment of the nanoscale physicochemical properties of mucins that are stored in a highly condensed state in the lumen of mucin granules is challenging and requires novel experimental approaches.

Small molecule probes are viable tools for reporting on the properties of various environments. Notably, fluorescent rotors are well-established viscometers that are used to gauge the viscosity of many biological systems^9,10^. In most cases, these viscometers undergo an internal rotation/twisting, producing a set of conformations that typically have different photophysical properties. Importantly, the rotation in the excited state is known to alter the fluorescence lifetime, which is one of the most significant photophysical properties. Since the lifetimes of fluorophores are independent of concentration, photobleaching, absorption, and excitation intensity, they are used for the unambiguous assessment of microviscosity^11–13^. Fluorescence lifetime imaging microscopy (FLIM), which provides images that are based on differences in excited state decays, allows for excellent sensitivity and high spatial resolution; thus, FLIM is an ideal tool for cellular and subcellular studies^11–13^. As such, fluorescent probes that could permeate the cell membrane, accumulate inside mucin granules, and whose lifetimes are sensitive to intragranular viscosity fluctuations would be a significant addition to the diagnostic/analytical tools of chemical biology.

## Results and Discussion

**Figure 1:**
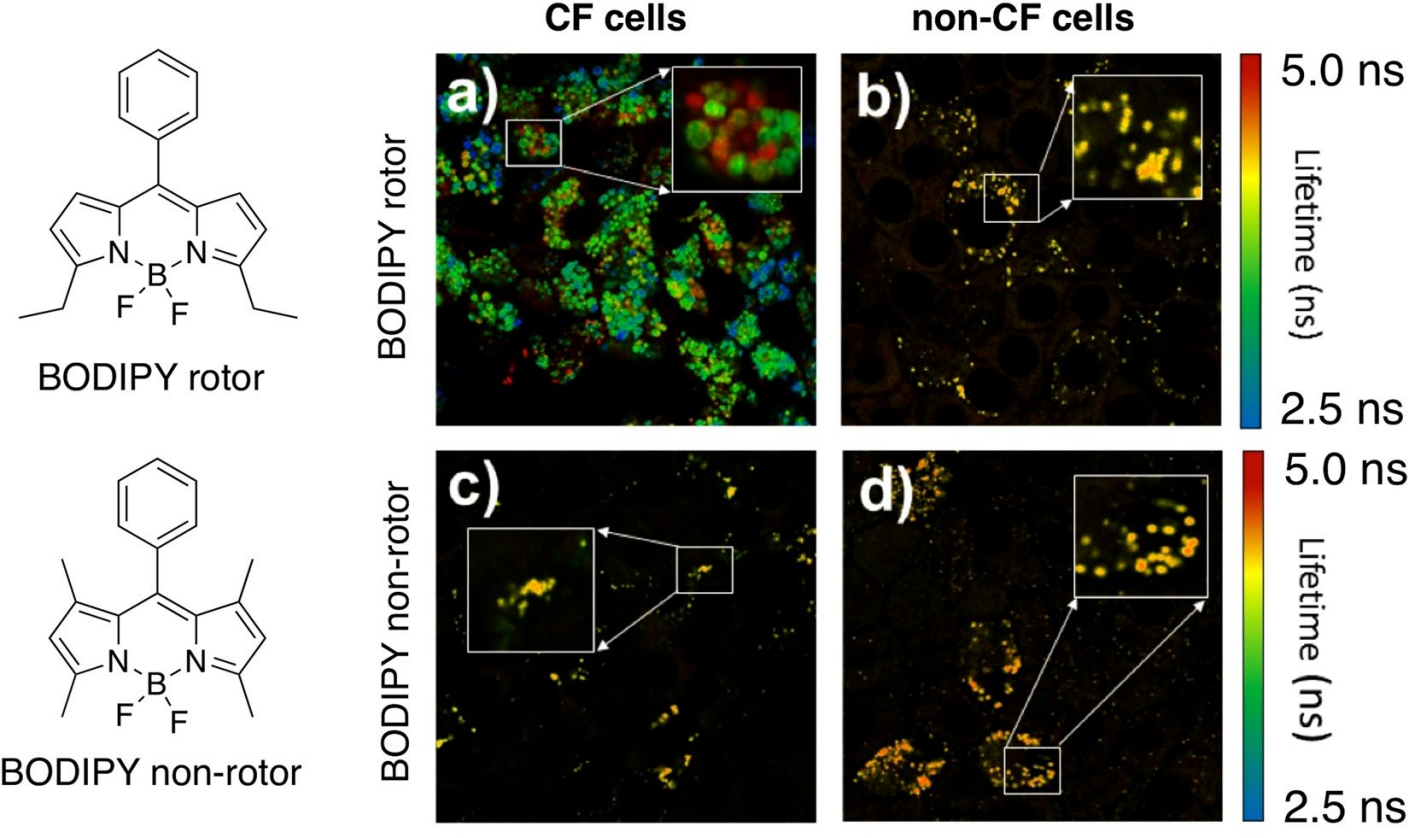
Structures of the dyes and FLIM images. FLIM images of the BODIPY rotor and non-rotor dyes incubated with CF (**a** and **c**) and non-CF (**b** and **d**) cells. Images scales are 80 × 80 µm. Insets: examples of high-resolution scans of the highlighted regions of interest.

BODIPY dyes are among the most versatile fluorophores that have been used as sensors and environmental probes for numerous applications, especially in biological and biomedical fields^14,15^. BODIPY-based viscometers proved to be useful in assessing the viscosity of various types of biological media, including membranes, tissues, and cellular environments^16–22^. In the course of our studies on developing BODIPY-based viscometers^16,17^, we discovered that a simple BODIPY dye (Fig. 1, where the rotation of the phenyl group around the BODIPY core is sensitive to the viscosity of the surrounding media, *i*.*e*., BODIPY rotor) showed appreciable membrane permeability and accumulation inside mucin granules. Here, we present the results on the use of BODIPY rotor to probe the viscosity of the intragranular mucin matrix of differentiated primary cultures of human bronchial epithelial cells from non-CF and CF patients (carrying the dominant CF mutation (F508del/F508), which affects 70 % of all CF patients worldwide^23^), which to the best of our knowledge, is the first example of a small molecule viscometer that is capable of reporting on the viscosity of intact mucin granule matrices in live cells.

In order to ensure that the fluorescence lifetimes of the BODIPY rotor were only related to the viscosity of its environment, and not to any other physical property of the media, such as polarity and pH, several control experiments were performed (Supplementary Figs 1–3; Supplementary Table 1). Specifically, the absorption and emission maxima appeared to be independent of the viscosity of glycerol-water mixtures (Supplementary Fig. 1; Supplementary Table 1). No changes in the fluorescence lifetimes were noted within the 5.5–8.5 pH range of the aqueous media or in organic solvents of various polarities (Supplementary Fig. 2). Importantly, at viscosities over 20 cP, such as those reported for mucus, *i*.*e*., significantly higher than 20 cP^6–8^, the fluorescence lifetimes of the BODIPY rotor linearly correlated with the viscosity of the media, and the relationship could be described by a modified Förster-Hoffmann equation (Supplementary Fig. 3), making this dye a reliable reporter for the range of apparent viscosities that might be expected for the intragranular matrix. In addition, we examined the behavior of BODIPY non-rotor (Fig. 1, where the rotation of the phenyl group around the BODIPY core is not possible due to the presence of two methyl groups) to demonstrate that no significant changes in the fluorescence lifetime occurred upon variation of viscosity, pH, or solvent polarity (Supplementary Figs 1–3; Supplementary Table 2).

To determine the viability of the BODIPY rotor (Fig. 1) as a mucin-specific viscometer, differentiated, primary cultures of bronchial epithelial cells from CF patients (F508del/F508del), were chosen.

Upon incubation with CF cells (Fig. 1a), the fluorescence lifetime of BODIPY rotor appeared to vary over a broad range (*i*.*e*., 2.5 to 5.0 ns), while minimal variation in the fluorescence lifetime was noted when BODIPY rotor was incubated with non-CF cells (Fig. 1b). Importantly, BODIPY non-rotor did not display any significant variations in fluorescence lifetime in CF or non-CF cells (Fig. 1c and d, respectively). These results strongly indicated that BODIPY rotor could potentially differentiate between healthy and diseased cells. It is also of interest to note that both the rotor and non-rotor showed good cell permeability and accumulation in granule lumen. Overall, these results suggested that viscometers based on BODIPY scaffolds could be used as intragranular probes for mucin matrix viscosity.

**Figure 2:**
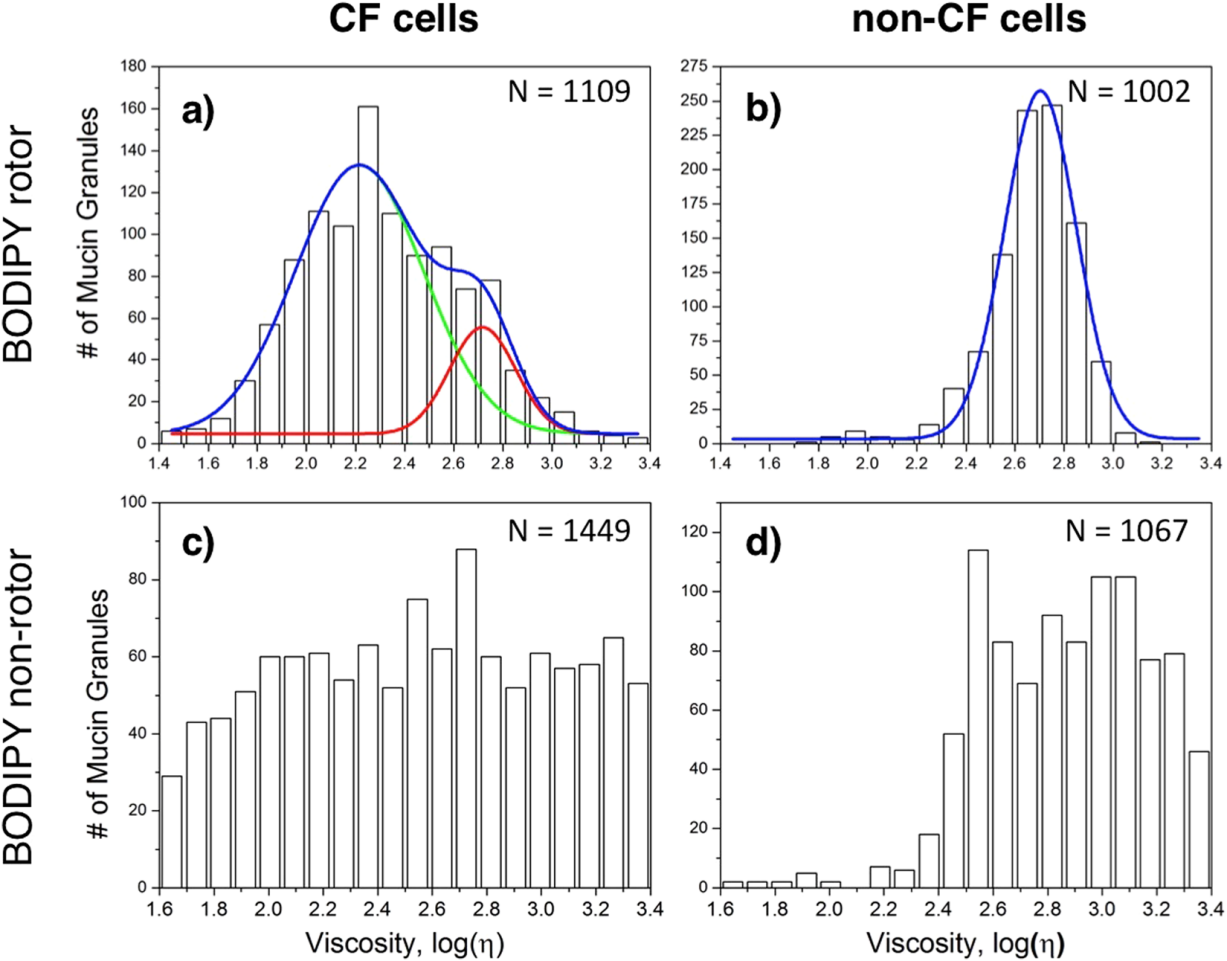
Viscosity of intragranular mucin matrix. The viscosity distribution of intragranular mucin matrix in CF (**a** and **c**) and non-CF (**b** and **d**) cells using rotor and non-rotor BODIPY dyes. The analysis was performed in three independent experiments with a total of 10–12 images analyzed per set. The number of granules (N) analyzed in each case is given at the right top corner of each panel. Blue, green and red lines are fits: blue – overall fits, green and red lines are fits for lower and higher viscosity populations.

In general, FLIM images of epithelial cells are highly dimensional and visually complex, which means that their analysis using conventional segmentation techniques could be time consuming. Moreover, image analysis is strongly dependent on user-based threshold parameters, which potentially could lead to user-biased results. Here, since each of the acquired FLIM images contained hundreds of mucin granule candidates, we chose to apply a machine-learning algorithm to segment the image and isolate individual mucin granules for high-throughput data analysis (see Supplementary Fig. 4 and online methods for details). Based on such assessment, the intragranular viscosity of a large population of individual mucin granules in airway epithelial cells collected from non-CF subjects and CF patients was determined (Fig. 2). The viscosity distributions revealed that non-CF cells had a single population of mucin granules with a viscosity centered around 520 cP (*i*.*e*., 521 ± 28 cP). Unexpectedly, the CF cells showed the presence of two populations of mucin granules. The smaller population had a viscosity of *ca*. 500 cP (*i*.*e*., 501 ± 46 cP), which was similar to that of the non-CF cells. However, the larger population had a lower viscosity around 160 cP (*i*.*e*., 164 ± 11 cP).

It should be noted that clusters/aggregates of mucin granules are commonly found in goblet cells of a variety of tissues^24–27^, thus the viscosity variations within the aggregates have also been analyzed. Specifically, we have performed comparative analysis of granule clusters and individual granules (see Supplementary Fig. 5). The results indicated that there were no appreciable differences between viscosities of mucin matrix of the individual granules and the granule’s aggregates.

Albeit preliminary, these results strongly suggest that the heterogeneity of mucin matrix viscosity in CF cells might be related to the disease, and provide an impetus for more detailed studies.

Notably, the size distributions of the mucin granules were found to be similar for both the CF and non-CF data sets (Supplementary Fig. 6). This confirmed that the mucin granules in CF and non-CF cells were not significantly different in regard to their maturation or swelling states. Therefore, the observed fluorescence lifetime variations between CF and non-CF cells were most likely the reflection of distinct viscosity distributions within their intragranular matrixes. Whether this variability is related to different degrees of mucin molecule packaging that could be affected, e.g., by alkalization of intraluminal pH in CF cells ^28,29^, will be determined in future studies.

## Conclusions

We demonstrated that BODIPY-rotor could probe intragranular viscosities of CF and non-CF cells. Importantly, two different populations of viscosities were identified in the CF granules as opposed to a single population of viscosities in non-CF granules. This indicates a heterogeneous nature of the CF granules, which might be related to the pathology. Overall, our results suggest that BODIPY-based viscometers could be viable tools for assessing the viscoelastic properties of mucin matrix within intact granules in live cells. Combining FLIM studies with such molecular viscometers should provide valuable insight into various stages of CF mucus pathogenesis, and potentially could aid in the development of efficient therapeutic approaches to combat the disease, for which no cure currently exists.

## Methods

### Cell culture

CF primary human airway epithelial cells (AEC), provided by the Respiratory Tissue and Cell Biobank of CRCHUM, were isolated from bronchial tissues collected from CF patients who underwent lung transplantation at CHUM hospital per approved ethical protocols and with written informed consent^30,31^. Non-CF human AEC, provided by the Primary Airway Cell Biobank (PACB) of Cystic Fibrosis  Translational Research Center, were from healthy individuals (without lung disease). Freshly isolated cells were seeded on flasks coated with Purecol (Cedarlane Laboratory, Burlington, ON, CA) and cultured in CnT-17 medium (CellnTec Advanced Cell Systems, Bern, CH) until 80% confluence was reached. Cells were then detached with trypsin solution, seeded on permeant filters (Corning, NY, USA) coated with collagen IV (Sigma-Aldrich, ON, Canada), and cultured in CnT-17 until confluency. Next, the medium was removed to create an air-liquid interface and the basolateral medium was replaced with differentiation medium (1:1 volume of BEGM and DMEM (Life Technologies, CA, USA) supplemented with 1.5 µg/mL BSA, 1 × 10^−7^ M retinoic acid, and 100 U/mL of penicillin-streptomycin every two days for at least 35 days to obtain highly differentiated cultures^30,31^.

### Viscosity of water-glycerol mixtures

Mixtures of various viscosities were prepared using commonly used glycerol-water mixtures^32–34^, by varying the volume fraction of a mixture of Milli-Q ultrapure water (EMD Millipore, MA, USA) and spectroscopic grade glycerol (Sigma-Aldrich, MA, USA) in 10% increments^35^.

### Steady state fluorescence measurements

All measurements were performed using 1.0 cm path length quartz cuvettes. Absorption measurements were acquired on a Varian Cary 50 Bio UV–Vis spectrophotometer (Agilent Technologies, CA, USA). Fluorescence measurements were performed on a Cary Eclipse spectrofluorometer (Agilent Technologies, CA, USA). Fluorescein (Sigma-Aldrich, MA, USA) in 0.1 M NaOH with a quantum yield of 0.95 was used as the standard for quantum yield calculations^36^. The quantum yield was determined using Eq. 1 as follows:

1 $${Q}=\,{{Q}}_{ref}\frac{{n}^{2}}{{n}_{ref}^{2}}\frac{I}{{I}_{ref}}\frac{1\,-\,{10}^{{A}_{ref}}}{1\,-{10}^{A}}\,$$

where, *Q* is the quantum yield of the sample, *n* is the index of refraction of the solvent the sample is in, *I* is the integrated intensity measured, and *A* is the absorbance of the sample. The remaining terms were the values from the reference standard.

### Fluorescence lifetime imaging microscopy

CF and non-CF primary human AEC were incubated with BODIPY rotor and non-rotor (2.3 µM each) for 30 min at 37 °C, under 5% CO_2_ atmosphere with mild agitation. Cells were washed twice for 2 minutes in HBSS w/o Ca^2+^ and Mg^2+^, mounted between coverslips, and examined using the Time-Correlated Single Photon Counting system. An MT-200 (PicoQuant, Berlin, Germany) confocal microscopy system  with a 60 × 1.2 NA Olympus water immersion objective and 50 µm pinhole was used with an Olympus IX71 inverted microscope with a piezoelectric scanning stage (Physik Instrumente, Karlsruhe, Germany) for all fluorescence imaging measurements and lifetime measurements. A PDL-470 (470 nm wavelength) laser operated at 20 MHz repetition rate by a PDL 828 “Sepia II” was used as the excitation source in all measurements. A 488 nm LP filter (Semrock, NY, USA) was used to remove the excitation from the collection. Symphotime V 4.2 (PicoQuant, Berlin, Germany) software was used to analyze and fit fluorescence lifetime decays. The time-dependent intensity decay curves were modeled as a series of exponential decays using Eq. 2 as follows:

2 $$I(t)=\sum _{i=1}^{M}{A}_{i}\exp (-t/{\tau }_{i})\,$$

where ${A}_{i}$, is the initial intensity, $t$ is the time in the lifetime trace, and ${\tau }_{i}$ is the characteristic fluorescence lifetime of the process. Fits used lifetime components such that the reduced ${\chi }^{2} \sim 1$. Fluorescence point spectra were collected by coupling an Ocean Optics USB2000 + (Ocean Optics, Florida, USA) to the MT200 microscope system.

### Image analysis

A machine-learning algorithm was applied to segment the image and isolate individual mucin granules. Individual granules were chosen over the aggregates to obtain uniform populations for the analysis. Specifically, the original FLIM images were mapped from lifetime space to viscosity space using the calibration curve. Trainable Weka Segmentation^37^ was used to train the classifier manually on several datasets. Once trained, the algorithm could be used to identify and isolate mucin granules in the images. We rejected all granules that did not fit into circularity criteria to assure that the observed responses were reported only from individual granules, rather than from their aggregates, which facilitated high-throughput data analysis and rapid analysis of thousands of granules (Supplementary Fig. 4).

## Electronic supplementary material

supporting information
