# Supplementary material for: Imaging viscosity of intragranular mucin matrix in cystic fibrosis cells
Source: Sci Rep. 2017 Dec 1;7:16761. doi: 10.1038/s41598-017-17037-2 (PMC5711894; doi:10.1038/s41598-017-17037-2)
Supplement: Supplementary file 1 — supporting information [file 41598_2017_17037_MOESM1_ESM.pdf]

SUPPLEMENTARY INFORMATION

Imaging viscosity of intragranular mucin matrix in cystic fibrosis cells

Sebastian Requena[1], Olga Ponomarchuk[2†], Marlius Castillo[3], Jonathan Rebik[3], Emmanuelle Brochiero[2,4], Julian Borejdo[1], Ignacy Gryczynski[1], Sergei V. Dzyuba[3]*, Zygmunt Gryczynski[1,5], Ryszard Grygorczyk[2,4]*, and Rafal Fudala[1]*

1. University of North Texas Health Science Center, Department of Microbiology, Immunology & Genetics, Fort Worth, TX, 76107, USA
2. Centre de Recherche du CHUM (CRCHUM), Université de Montréal, Montréal, Québec, H2X 0A9, Canada
3. Texas Christian University, Department of Chemistry and Biochemistry, Fort Worth, TX, 76129, USA
4. Département de Médecine, Université de Montréal, Montréal, Québec, H3T 1J4, Canada
5. Texas Christian University, Department of Physics and Astronomy, Fort Worth, TX, 76129, USA

* e-mail: ryszard.grygorczyk@umontreal.ca, s.dzyuba@tcu.edu, Rafal.Fudala@unthsc.edu

† visiting graduate student from Moscow State University, Russia

TABLE OF CONTENT

Synthesis Rotor and non-rotor BODIPY dyes…………………………….……………..…… 2

Supplementary Figure 1 Absorption and emission spectra of rotor and non-rotor BODIPY dye…..…………………………………………………………………........... 3

Supplementary Figure 2 The lifetime of rotor and non-rotor BODIPY dyes as a function of pH and polarity of the media…………………………..…………………..…….. 4

Supplementary Figure 3 The Förster-Hoffmann plot of the rotor and non-rotor BODIPY dyes……………………………………………………………………………….…... 5

Supplementary Figure 4 A flowchart of the image analysis process……………………… 6

Supplementary Figure 5 Images and viscosities of aggregated and individual mucin granules... 7

Supplementary Figure 6 Size distribution of mucin granules…….………………………... 8

Supplementary Table 1 Spectroscopic properties of the rotor BODIPY in water – glycerol mixture…………………………………………………………………….. 9

Supplementary Table 2 Spectroscopic properties of the non-rotor BODIPY in water – glycerol mixtures……………………………………………………………………………… 10

References ……………………………………………………………………………… 11

Synthesis of rotor and non-rotor BODIPY dyes

All chemicals and solvents used for the synthesis of BODIPY dyes were of the highest grade possible and used as received from commercial vendors. BODIPY rotor and non-rotor were prepared according to previously published procedure.[1]

Rotor BODIPY:[2]

^{1}H NMR (300MHz, $CDCl_3$): δ = 7.47 (m, 5H), 6.73 (d, *J* = 4.1 Hz, 2H), 6.34 (d, *J* = 4.1 Hz, 2H), 3.08 (q, *J* = 7.6 Hz, 4H), 1.34 (t, *J* = 7.6 Hz, 6H).

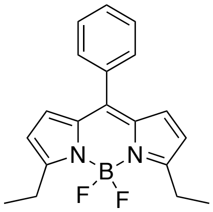

Non-rotor BODIPY:[3,4]

^{1}H NMR (300 MHz, $CDCl_3$): δ = 7.48 (m, 3H), 7.28 (m, 2H), 5.98 (s, 2H), 2.56 (s, 6H), 1.37 (s, 6H).

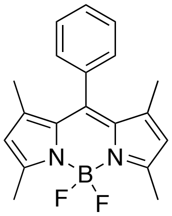

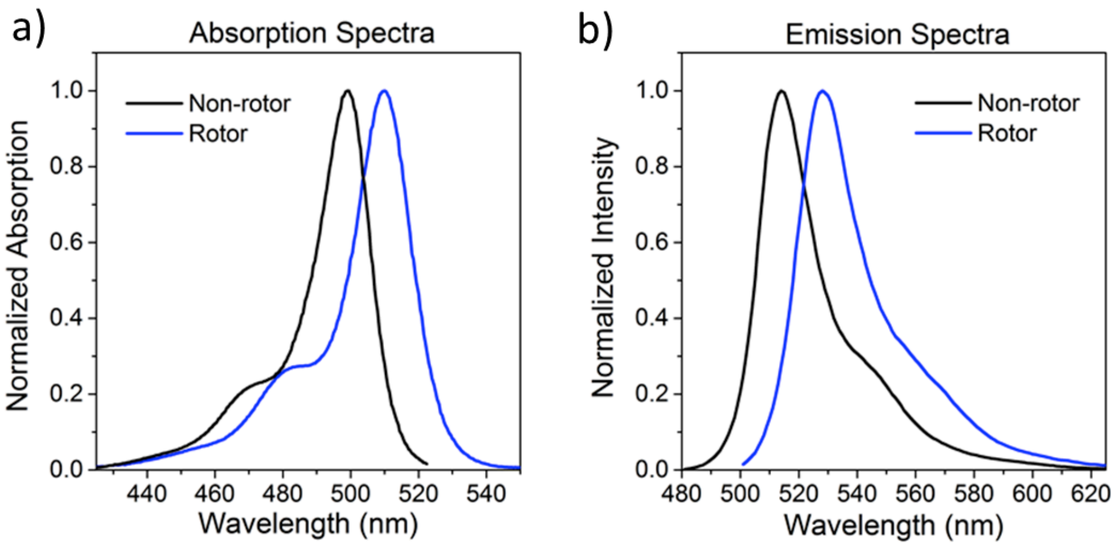

Supplementary Figure 1: Absorption (a) and emission (b) spectra of the rotor (blue) and non-rotor (black) BODIPY. Conditions: [BODIPY] = 2.3 μM, λ_{ex} = 470 nm; solvent – MeOH.

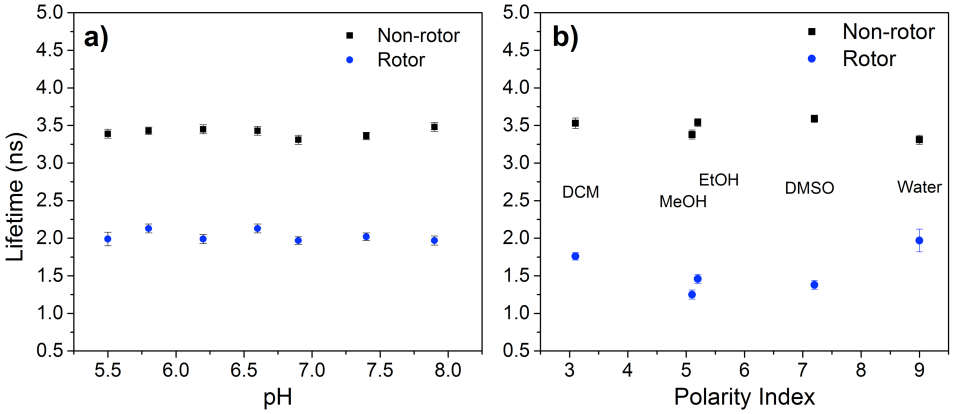

Supplementary Figure 2: The lifetime of rotor and non-rotor BODIPY dyes as a function of pH (a), and polarity (b) of the media.

The solvents are: dichloromethane (DCM), methanol (MeOH), ethanol (EtOH), dimethylsulfoxide (DMSO), and water. The data are reported as an average from three experiments ± SD.

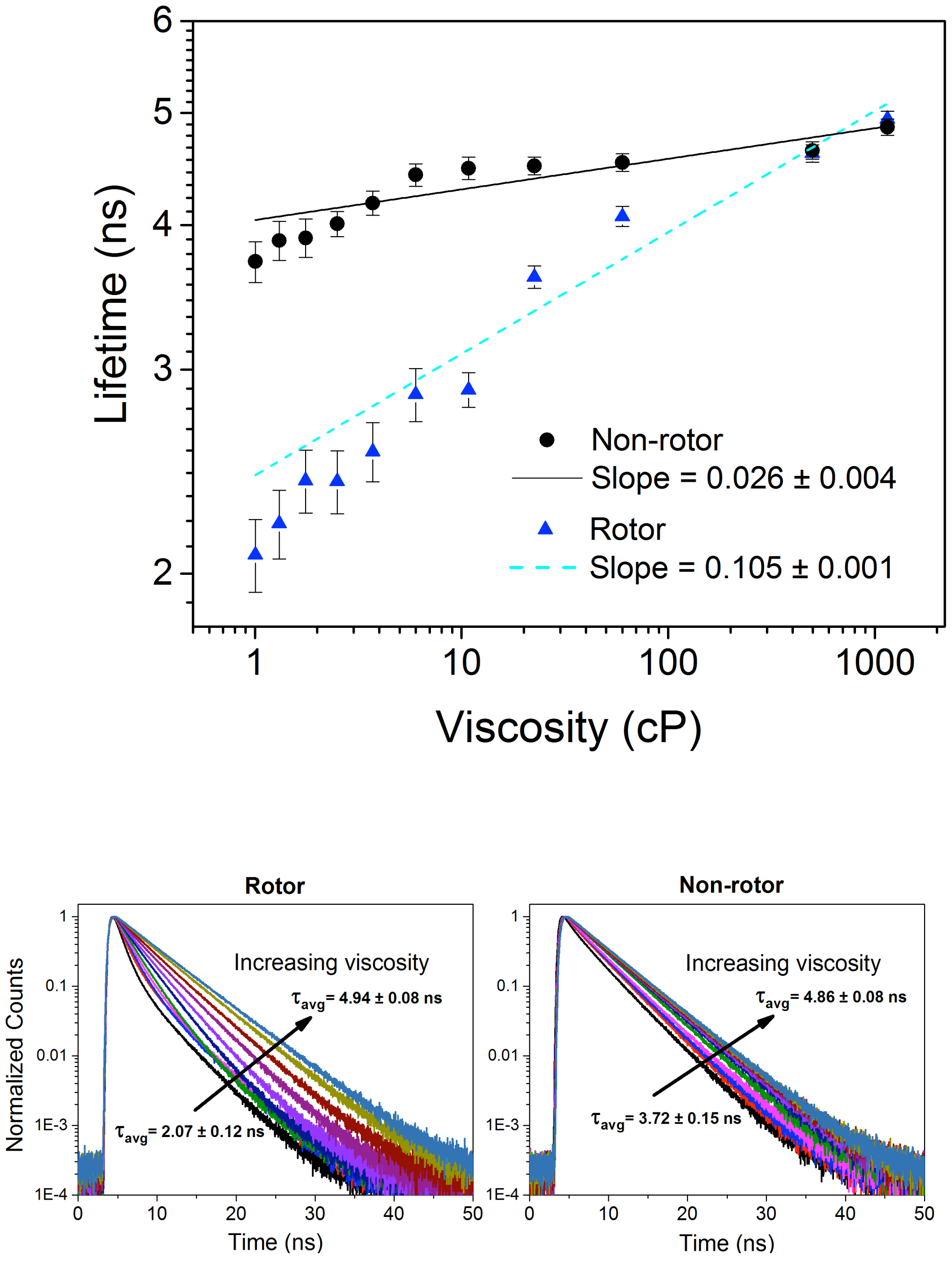

Supplementary Figure 3 The Förster-Hoffmann plot of the rotor and non-rotor BODIPY dyes (top) and normalized lifetimes of rotor and no-rotor BODIPY dyes as a function of media's viscosity (bottom).

Fluorescent measurements were performed in triplicates at 22°C.

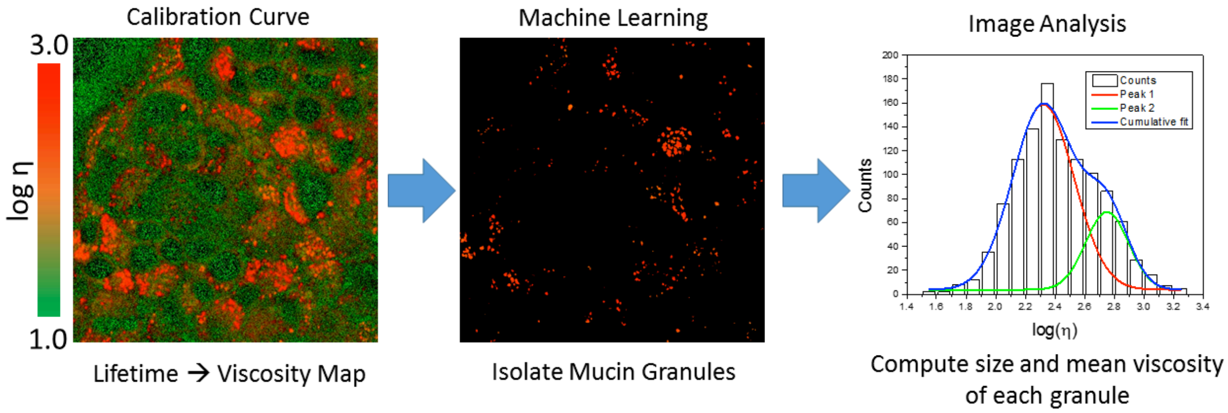

Supplementary Figure 4: A flowchart of the image analysis process. The fluorescence lifetime calibration curve is used to convert FLIM images to viscosity images, a machine learning algorithm segments the mucin granules and isolates them from the background. The images are analyzed to obtain the mean area and viscosity of each granule.

a)

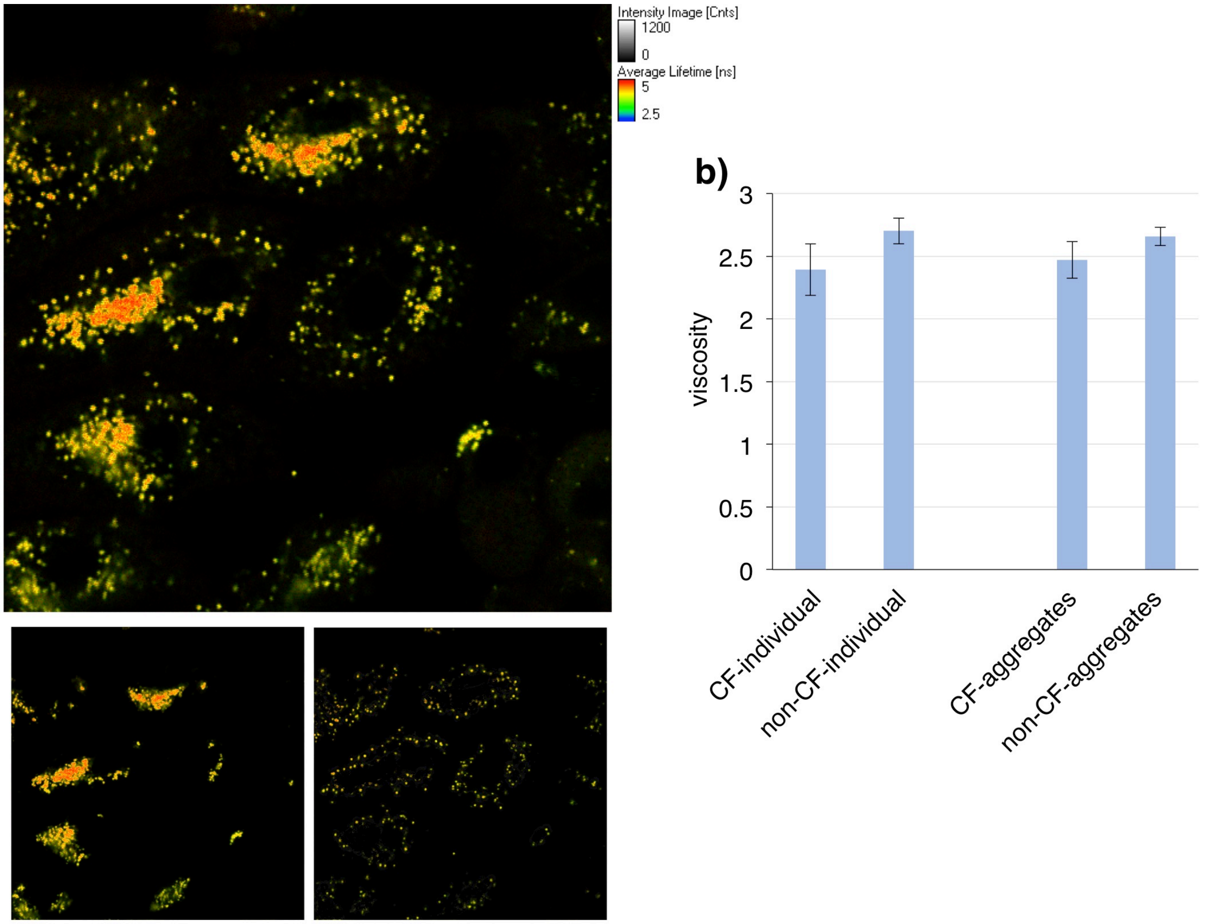

Supplementary Figure 5: Images and viscosities of aggregated and individual granules.

a) an image of aggregated and individual non-CF granules; the segmented/extracted images of the aggregates (left) and individual (right) granules are shown underneath;

b) viscosities of individual and aggregated granules (*ca.* 20 granules of each type were analyzed; results are given as averages ± SD).

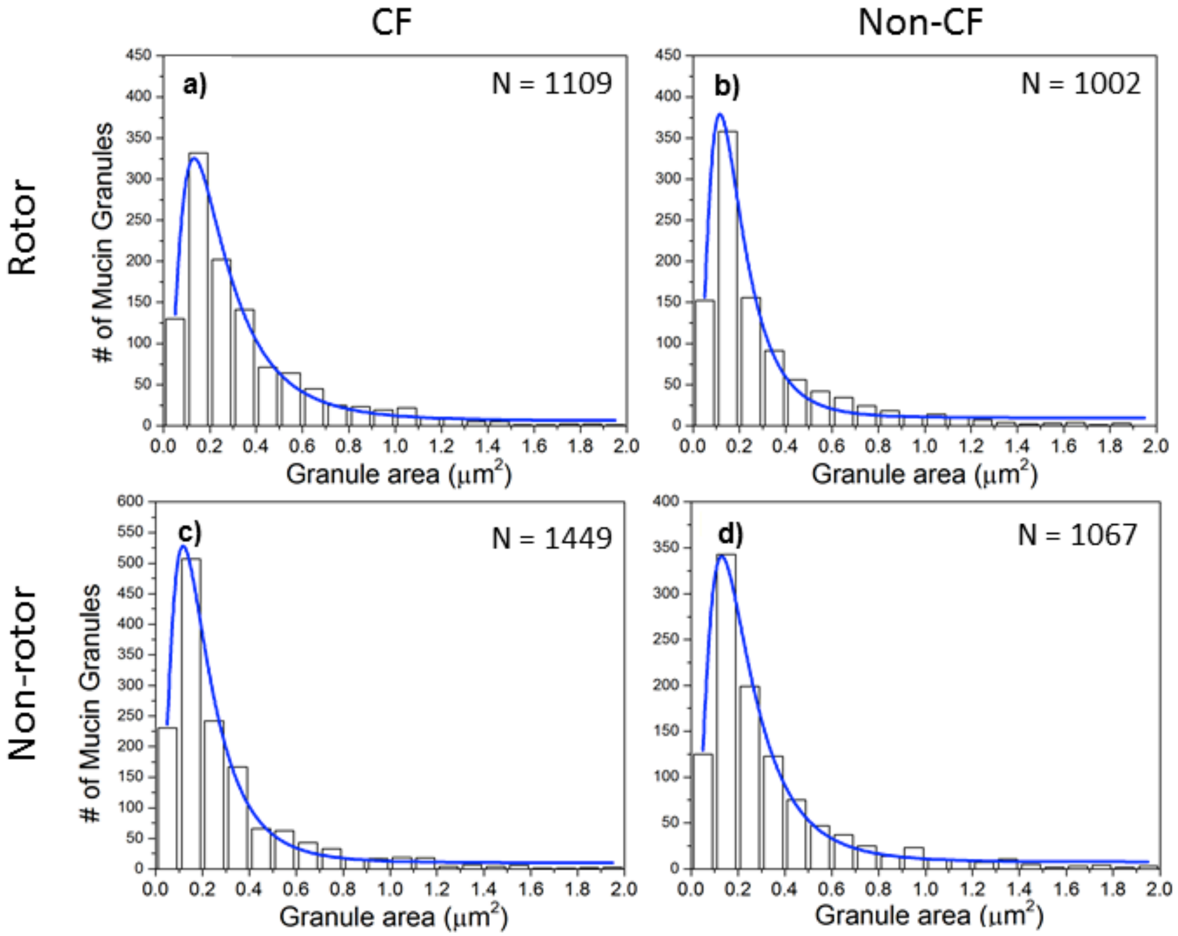

Supplementary Figure 6: Size distribution of mucin granules.

a) CF cells/rotor experiments; the diameter of the granules (total of 1109) is centered at 530 nm;

b) non-CF cells/rotor experiments: the diameter of the granules (total of 1002) is centered at 470 nm;

c) CF cells/non-rotor experiments: the diameter of the granules (total of 1449) is centered at 478 nm;

d) non-CF cells/non-rotor experiments the diameter of the granules (total of 1067) is centered at 511 nm.

Analysis was performed on three independent experiments with a total of 10-12 images analyzed per set.

Supplementary Table 1: Spectroscopic properties of the rotor BODIPY in water – glycerol mixtures.[a]

**Viscosity (cP)[b]**	**λ_{ab}^{max} (nm)[c]**	**λ_{em}^{max} (nm)[d]**	**τ_{avg} (ns)[e]**	***Q*[f]**
1150	512 ± 3	528 ± 3	4.94 ± 0.08	0.80 ± 0.01
219	512 ± 3	528 ± 3	4.61 ± 0.08	0.69 ± 0.01
60.1	512 ± 3	528 ± 3	4.07 ± 0.08	0.49 ± 0.01
22.5	512 ± 3	527 ± 3	3.61 ± 0.08	0.37 ± 0.01
10.5	512 ± 3	527 ± 3	2.88 ± 0.10	0.27 ± 0.02
6	511 ± 3	526 ± 3	2.86 ± 0.10	0.21 ± 0.02
3.72	512 ± 3	526 ± 3	2.55 ± 0.10	0.13 ± 0.02
2.5	511 ± 3	527 ± 3	2.40 ± 0.15	0.08 ± 0.03
1.76	511 ± 3	526 ± 3	2.41 ± 0.15	0.07 ± 0.03
1.31	512 ± 3	526 ± 3	2.21 ± 0.15	0.06 ± 0.03
1.0	511 ± 3	526 ± 3	2.08 ± 0.15	0.05 ± 0.03

[a] – all measurements were performed in triplicates and data is represented as the average ± the standard deviation; [b] – data is taken from ref. 5; [c] – the absorption maximum, [d] – the emission maximum; [e] – amplitude averaged fluorescent lifetime; [f] – quantum yield

Supplementary Table 2: Spectroscopic properties of the non-rotor BODIPY in water – glycerol mixtures.[a]

**Viscosity (cP)**[b]	**λ_{ab}^{max} (nm)**[c]	**λ_{em}^{max} (nm)**[d]	**τ_{avg} (ns)**[e]	***Q***[f]
1150	501 ± 3	515 ± 3	4.86 ± 0.06	0.81 ± 0.01
219	501 ± 3	515 ± 3	4.64 ± 0.08	0.70 ± 0.01
60.1	501 ± 3	515 ± 3	4.53 ± 0.08	0.55 ± 0.01
22.5	501 ± 3	514 ± 3	4.50 ± 0.08	0.49 ± 0.01
10.5	502 ± 3	514 ± 3	4.48 ± 0.10	0.46 ± 0.01
6	502 ± 3	512 ± 3	4.42 ± 0.10	0.45 ± 0.01
3.72	501 ± 3	513 ± 3	4.18 ± 0.10	0.42 ± 0.01
2.5	501 ± 3	511 ± 3	4.01 ± 0.10	0.41 ± 0.01
1.76	501 ± 3	511 ± 3	3.90 ± 0.10	0.39 ± 0.01
1.31	501 ± 3	511 ± 3	3.88 ± 0.15	0.38 ± 0.01
1.0	501 ± 3	511 ± 3	3.72 ± 0.15	0.31 ± 0.02

[a] – all measurements were performed in triplicates and data is represented as the average ± the standard deviation; [b] – data is taken from ref. 5; [c] – the absorption maximum, [d] – the emission maximum; [e] – amplitude averaged fluorescent lifetime; [f] – quantum yield

References

1. Jameson, L. P. & Dzyuba, S. V. Expeditious, mechanochemical synthesis of BODIPY dyes. *Beilstein J. Org. Chem.* **9**, 786–790 (2013).

2. Smith, N. W., Alonso, A., Brown, C. M. & S. V. Dzyuba, Triazole-containing BODIPY dyes as novel fluorescent probes for soluble oligomers of amyloid Aβ1-42 peptide. *Biochem. Biophys. Res. Commun.* **391**, 1455–1458 (2010).

3. Chen, Y., Zhao, J., Guo, H. & Xie, L. Geometry relaxation-induced large Stokes shift in red-emitting borondipyrromethenes (BODIPY) and applications in fluorescent thiol probes. *J. Org. Chem.* **77**, 2192–2206 (2012).

4. Wu, W., Guo, H., Wu, W., Ji, S. & Zhao, J. Organic triplet sensitizer library derived from a single chromophore (BODIPY) with long-lived triplet excited state for triplet-triplet annihilation based upconversion. *J. Org. Chem.* **76**, 7056–7064 (2011).

5. Shankar, P.N. & Kumar, M. Experimental determination of the kinematic viscosity of glycerol-water mixtures. *Proc. Royal Soc. A* **444**, 573–581 (1994).
